# Cellular Uptake and Intracellular Phosphorylation of GS-441524: Implications for Its Effectiveness against COVID-19

**DOI:** 10.3390/v13071369

**Published:** 2021-07-14

**Authors:** Henrik Berg Rasmussen, Gesche Jürgens, Ragnar Thomsen, Olivier Taboureau, Kornelius Zeth, Poul Erik Hansen, Peter Riis Hansen

**Affiliations:** 1 Institute of Biological Psychiatry, Mental Health Centre Sct. Hans, DK-4000 Roskilde, Denmark; 2 Department of Science and Environment, Roskilde University Center, DK-4000 Roskilde, Denmark; kzeth@ruc.dk (K.Z.); poulerik@ruc.dk (P.E.H.); 3 Clinical Pharmacology Unit, Zealand University Hospital, DK-4000 Roskilde, Denmark; gju@regionsjaelland.dk; 4 Section of Forensic Chemistry, Department of Forensic Medicine, Faculty of Health Sciences, University of Copenhagen, DK-2100 Copenhagen, Denmark; ragnar.thomsen@sund.ku.dk; 5 INSERM U1133, CNRS UMR 8251, Université de Paris, F-75013 Paris, France; olivier.taboureau@u-paris.fr; 6 Department of Cardiology, Herlev and Gentofte Hospital, DK-2900 Hellerup, Denmark; peter.riis.hansen@regionh.dk

**Keywords:** GS-441524, adenosine analogs, COVID-19, adenosine transporters, adenosine kinase, adenosine levels

## Abstract

GS-441524 is an adenosine analog and the parent nucleoside of the prodrug remdesivir, which has received emergency approval for treatment of COVID-19. Recently, GS-441524 has been proposed to be effective in the treatment of COVID-19, perhaps even being superior to remdesivir for treatment of this disease. Evaluation of the clinical effectiveness of GS-441524 requires understanding of its uptake and intracellular conversion to GS-441524 triphosphate, the active antiviral substance. We here discuss the potential impact of these pharmacokinetic steps of GS-441524 on the formation of its active antiviral substance and effectiveness for treatment of COVID-19. Available protein expression data suggest that several adenosine transporters are expressed at only low levels in the epithelial cells lining the alveoli in the lungs, i.e., the alveolar cells or pneumocytes from healthy lungs. This may limit uptake of GS-441524. Importantly, cellular uptake of GS-441524 may be reduced during hypoxia and inflammation due to decreased expression of adenosine transporters. Similarly, hypoxia and inflammation may lead to reduced expression of adenosine kinase, which is believed to convert GS-441524 to GS-441524 monophosphate, the perceived rate-limiting step in the intracellular formation of GS-441524 triphosphate. Moreover, increases in extracellular and intracellular levels of adenosine, which may occur during critical illnesses, has the potential to competitively decrease cellular uptake and phosphorylation of GS-441524. Taken together, tissue hypoxia and severe inflammation in COVID-19 may lead to reduced uptake and phosphorylation of GS-441524 with lowered therapeutic effectiveness as a potential outcome. Hypoxia may be particularly critical to the ability of GS-441524 to eliminate SARS-CoV-2 from tissues with low basal expression of adenosine transporters, such as alveolar cells. This knowledge may also be relevant to treatments with other antiviral adenosine analogs and anticancer adenosine analogs as well.

## 1. Background

Despite the emergence of vaccines for prevention of COVID-19, there is a continuing need for potent antiviral agents in the treatment of the disease. This partly reflects that COVID-19 vaccines do not protect all individuals and that an appreciable number of individuals may refuse to receive the vaccine or be ineligible for vaccination due to pre-existing medical conditions.

Remdesivir was originally developed for treatment of infections with RNA viruses possessing the potential to develop into pandemics such as Ebola virus disease [1], and received emergency approval for treatment of COVID-19 based on findings that it decreased mortality and shortened hospital stays [2]. However, subsequent data did not confirm the beneficial effects of remdesivir, which led the most recent WHO guidance on drugs for treatment of COVID-19 to recommend against use of this drug in hospitalized patients [3].

**Figure 1:**
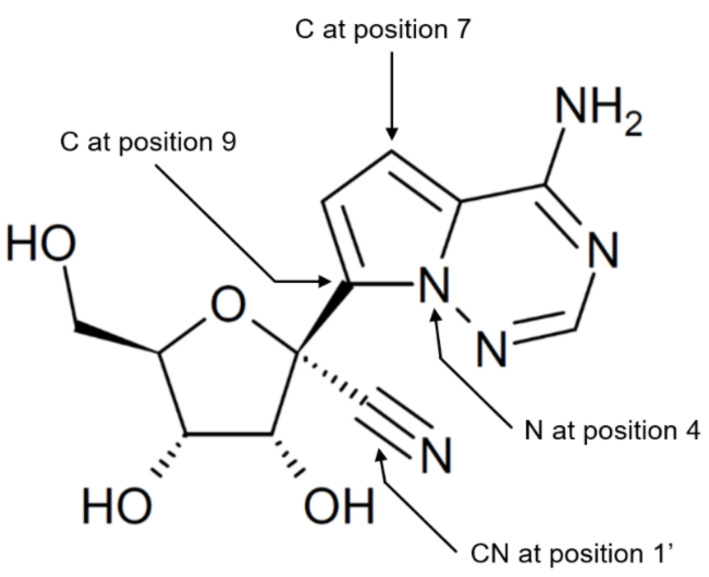
Chemical structure of GS-441524. GS-441524 is an adenosine analog that differs from adenosine by having N instead of C at position 4, and C instead of N at positions 7 and 9 in the adenine ring, and by having CN (cyano) group at position 1′ in the ribose moiety.

Remdesivir belongs to the group of ProTides, being a nucleoside analog prodrug with masking of the hydroxyls on its phosphoric acid group to enhance transmembrane diffusion and intracellular accumulation in a manner that largely is independent of nucleoside transporters [1,4,5]. The nucleoside analog in remdesivir is GS-441524, which is a derivative of adenosine (Figure 1). Remdesivir is hydrolyzed to GS-441524 monophosphate after cellular uptake. The phosphate group on GS-441524 serves to overcome the first and perceived rate-limiting phosphorylation step in the formation of GS-441524 triphosphate, the active antiviral agent.

The pharmacokinetics of GS-441624 differ from those of remdesivir in several respects. Lacking the hydrophobic prodrug moieties and the phosphate group of remdesivir, cellular uptake of GS-441524 is probably to a large extent dependent on nucleoside transporters, which can be saturated at high substrate concentrations [6]. Moreover, intracellular formation of GS-441524 triphosphate may be reduced due to the absence of the monophosphate group in GS-441524.

Several in vitro studies have reported the high ability of GS-441524 to inhibit SARS-CoV-2 [7,8]. Based on different procedures for viral quantification, one of these studies reported a half-maximal inhibitory concentration (IC_50_) value of 0.47 μM for the anti-SARS-CoV-2 activity of GS-441524 in Vero cells, and IC_50_ values of 0.62 and 1.09 μM for its ability to inhibit this virus in the Calu-3 2B4 human lung cancer cell line [8]. Another study found that GS-441524 inhibited SARS-CoV-2 with an IC_50_ value of 0.70 μM in Vero cells, and determined IC_50_ values of 3.21 and 3.62 μM for the anti-SARS-CoV-2 activity of this agent in Calu-3 cells and Caco-2 cells, respectively [7]. Of note, the IC_50_ values of 0.47 and 0.70 μM produced by GS-441524 were lower than the IC_50_ values of 1.35, 1.49 and 1.65 μM observed for the anti-SARS-CoV-2 activity of remdesivir in Vero cells [7,8]. Besides effectively inhibiting SARS-CoV-2, GS-441524 has been found to inhibit SARS-CoV and MERS-CoV with IC_50_ values of about 0.86 μM in human airway epithelial cells [9].

Previously, GS-441524 was found to be highly effective in the treatment of feline infectious peritonitis caused by a feline coronavirus [10]. This agent was also reported to significantly inhibit SARS-CoV-2 in a mouse model [7]. Importantly, GS-441524 appears to possess a favorable safety profile [10], thus potentially allowing for increased dosing. The relatively simple structure of GS-441524 makes it a candidate for mass production. The synthesis of remdesivir is more complex and a lengthy process [7,11] but a procedure for faster manufacture of this agent has been proposed [12].

Recently, GS-441524 was proposed as a candidate for treatment of COVID-19, potentially being more effective than remdesivir against this disease [11]. We here discuss cellular uptake and intracellular phosphorylation of GS-441524 and based on this assess its effectiveness for treatment of COVID-19.

## 2. Transporters Implicated in Transport of Adenosine and Adenosine Analogs

Efficiency of the transport of antiviral and anticancer nucleoside analogs across cell membranes may be important in determining their intracellular concentrations and treatment outcomes. Since nucleosides generally are hydrophilic, and thus possess low ability to cross cell membranes by diffusion, their transmembrane passage is to a major extent dependent on two families of specialized membrane molecules, namely, the concentrative nucleoside transporter (CNT) family with the members CNT1-3 and the family of equilibrative nucleoside transporters (ENTs) consisting of ENT1-4 [13,14,15]. The family of CNTs are obligatory inward transporters that mediate cellular influx against concentration gradients, with CNT2 and CNT3 being the most important members of this family in the uptake of adenosine [14,16]. Apparently, CNT3 has higher affinity for adenosine than CNT2 [14]. ENTs facilitate molecular diffusion across cell membranes with the potential to act as bidirectional transporters, and appear to play a major role in maintaining adenosine homeostasis with ENT1, possessing higher affinity for adenosine and probably being more important in the transport of this nucleoside than ENT2 [14,16]. While CNTs may be involved in specialized functions, including crosstalk with nucleotide receptors and signal transduction, the major role of the ENTs, which are expressed across a broader range of types of cells and tissues than the CNTs, may be limited to maintaining nucleoside homeostasis [14]. Both ENT1 and ENT2 have significantly lower affinities for adenosine than CNT2 and CNT3, but this is probably, in part, compensated by phosphorylation of adenosine inside the cells, which lowers intracellular adenosine levels and creates a transmembrane concentration gradient favoring influx of adenosine [16].

Cellular uptake of a selection of adenosine and guanosine analogs, predominantly analogs in clinical use, appears largely to be dependent upon the same transporters as those responsible for adenosine uptake. The XLogP3 values of these nucleoside analogs, as measures of their hydrophobicity, ranged from −2.3 to 0.9, thus being comparable to the values of adenosine and GS-441524 of −1.1 and 1.4, respectively (Table 1). Hence, diffusion across cell membranes is probably not the major mechanism of transport of clinically used adenosine analogs into cells, since this type of transport is slow for hydrophilic compounds, consistent with the notion that transmembrane passage of adenosine by free diffusion is negligible [16,17]. However, other nucleoside analogs, including those with alkyl, silylate, and acyl groups, are significantly more hydrophobic [18]. For example, 2′,5′-di-O-trityluridine has an XLogP3 value of 7.9. In support of a limited role of diffusion in the translocation of hydrophilic adenosine analogs across cell membranes, influx of tecadenoson with an XLogP3 value of −0.3 has been found to be slow in *Xenopus* oocytes compared to *Xenopus* oocytes expressing human ENT1 [19]. A previous study of sulfamoyl adenosine analogs also showed that higher hydrophobicity lead to higher accumulation in bacteria, and that lack of antibacterial activity was linked with low hydrophobicity [20].

Kinetic parameter values for the influx of adenosine and adenosine analogs mediated by ENT1 and ENT2, probably the major transporters in the transmembrane passage of this group of compounds [21,22], are shown in Table 2. Although comparison of these values, which derive from different studies, may be influenced by use of different experimental methods, the reported Michaelis constant (K_m_) values appear to be higher for the adenosine analogs than for adenosine, suggesting that ENT1 and ENT2 prefer adenosine over most adenosine analogs. There may also be relatively large differences in the affinity of different adenosine analogs for ENT1 and ENT2.

**Table 1:** Pentose group-containing purine nucleoside analogs and their transporters.

Adenosine Analog	Indication and/or Activity	Transporter ^1^	XLogP3 ^2^	Reference
Fludarabine phosphate ^3^	Cancer (chemotherapy)	ENT1, ENT2, CNT3	−0.6	[23,24]
Clofarabine	Cancer (chemotherapy)	ENT1, ENT2, CNT2, CNT3	0.9	[23]
Nelarabine ^4^	Cancer (chemotherapy)	ENT1, ENT2	−0.7	[25]
Forodesine (immucillin H)	Cancer (chemotherapy)	ENT1, ENT2	−2.3	[23]
Cordycepin (3′-deoxyadenosine)	Potential antineoplastic activity	ENT1, ENT2	−1.2	[26]
Cladribine (2-chlorodeoxyadenosine)	Cancer (chemotherapy)	ENT1, ENT2, CNT3	0.8	[23,27]
7-Deazaadenosine (tubercidin)	Antibiotic and cytostatic	Probably, in particular ENT1	−1.3	[28]
8-Chloro-adenosine	Potential antineoplastic activity	Probably, in particular ENT1	−0.1	[29]
Pentostatin	Cancer (chemotherapy)	ENT1, ENT2	−2.1	[30]
Tecadenoson	A1 adenosine receptor agonist developed for treatment of paroxysmal supraventricular tachycardia	Primarily ENT1	−0.3	[19]

^1^ ENT1: solute carrier family 29 member 1, SLC29A1. ENT2: solute carrier family 29 member 2, SLC29A2. CNT2: solute carrier family 28 member 2, SLC28A2. CNT3: solute carrier family 28 member 3, SLC28A3. ^2^ From PubChem, which also reports XlogP3 values for adenosine and GS-441524 of −1.1 and −1.4, respectively [31]. ^3^ Rapidly dephosphorylated in plasma to form fludarabine. ^4^ Rapidly demethylated to ara-Guanosine (ara-G) after intravenous administration.

Reported levels of expression of the transporters implicated in the translocation of adenosine and adenosine analogs across cell membranes in the human respiratory tract are shown in Table 3. Apparently, there is a higher level of expression of these transporters in the epithelial cells of upper airways than those in lower airways that line the alveoli and consist of the alveolar type 1 and 2 cells. Moreover, the expression of the transporters translocating adenosine and adenosine analogs across cell membranes appears to be low or absent in alveolar cells, except for ENT2 with a medium-level of expression [33]. Of note, ENT1, possibly the most important of the adenosine transporters in maintaining metabolic homeostasis of adenosine [14], is expressed at only low levels in alveolar cells [33]. Since SARS-CoV-2 primarily seems to infect ciliated cells in the upper airways and alveolar type 2 cells in the lower airways [34], effectiveness of GS-441524 in the treatment of COVID-19 may require that these specific cells express the molecules implicated in the uptake of adenosine analogs at sufficiently high levels.

## 3. Extracellular Adenosine Levels

Adenosine is an endogenous molecule that has a half-life of a few seconds, and therefore acts locally. Extracellular adenosine binds to receptors on cell surfaces to regulate functions in tissues, organs and organ systems, including the immune system where it has suppressive effect [36,37]. Levels of local extracellular adenosine may be important in determining cellular uptake of GS-441524 by competition for the nucleoside transporters. This has been observed in in vitro systems where adenosine inhibited the cellular uptake of tubercidin, an adenosine analog, and 6-methoxypurine arabinoside, with inhibitory constant (K_i_) values of 38 µM [38] and 134 µM [39], respectively.

Levels of adenosine in body fluids have been suggested to be in the range from 30 to 300 nM under physiological conditions, with metabolism and cellular uptake playing major roles in maintaining adenosine homeostasis [40]. A higher physiological concentration interval of adenosine from 400 to 800 nM in plasma was adopted by a recent study [41]. These differences probably both reflect use of different analytical procedures and variation among study subjects.

The extracellular level of adenosine is increased in a variety of diseases, in particular those characterized by systemic inflammation, metabolic stress and critical illness, probably reflecting a role of this nucleoside in protecting tissues from hypoxia and injury [42,43,44]. For example, ischemia has been found to lead to receptor level concentrations of adenosine up to 30 μM in rat hippocampal slices, i.e., 100–150-fold higher levels than those observed under normal conditions [45], and in ischemic rat brains extracellular adenosine levels of up to 40 μM have been reported [46]. Moreover, plasma adenosine levels of 3.9 and 8.4 μM have been detected in patients with severe sepsis and septic shock, respectively, which markedly exceeds the levels of 0.8 μM in healthy control subjects, and 1.1 µM in patients with hemorrhagic traumatic shock [47]. Additionally, both extracellular and intracellular levels of adenosine have been reported to increase 10–20-fold in inflammation during hypoxia and in hypoxic tumor microenvironments [48]. In line with this, adenosine levels ranging from 22.03 to 210.84 μM, with a mean of 86.95 μM, have been found in bone marrow aspirates from patients with active and relapsed multiple myeloma, while bone marrow adenosine levels were markedly lower in other hematological malignancies and in asymptomatic plasma cell disorders that may develop into multiple myeloma [49].

It is likely that extracellular adenosine is also produced in excessive amounts during viral infections. Importantly, interferon-α is released during such infections [50] and has been found to upregulate the in vitro expression of the ecto-5′-nucleotidase CD73 on endothelial cells, which converts adenosine monophosphate to adenosine [51]. Further, infection of cultures of human bronchial epithelial cells with recombinant respiratory syncytial virus increased goblet cell formation and adenosine concentrations in fluid sampled from the air-cell interface [52]. Infection of mice with influenza A virus significantly increased adenosine concentration in bronchoalveolar lavage fluid [53]. Along this line, higher levels of adenosine have been reported in bronchoalveolar lavage fluid from smokers and asthma patients than in normal subjects [54].

High extracellular levels of adenosine during hypoxia and inflammation may reflect increased production of this nucleoside due to increased cellular degradation of adenosine triphosphate, which is present at levels of 4–8 µM intracellularly [55]. Decreased expression of ENT1 and ENT2 in hypoxia [56] and decrease in the ENT2 expression mediated by inflammatory cytokines or lipopolysaccharide [57] may also lead to increased levels of extracellular adenosine. Most likely, increased extracellular levels of adenosine combined with decreased expression of adenosine transporters during hypoxia and inflammation may significantly reduce cellular uptake of GS-441524, potentially leading to low therapeutic effect, particularly in tissues with low basal expression of these transporters.

Besides pathological conditions, several pharmaceutical agents have the ability to alter extracellular concentrations of adenosine. For example, the antiplatelet agents dipyridamole and dilazep, both inhibitors of ENT1 and ENT2 [58], have been reported to increase plasma adenosine levels by almost two-fold [59]. Other drugs with the ability to increase plasma adenosine levels include cyclosporin and tacrolimus [60].

The reported K_i_ values for the adenosine-mediated inhibition of the transport of tubercidin and methoxypurine arabinoside of 38 μM [28] and 134 μM [39], respectively, both were above the adenosine level of 8.4 µM in septic shock [47] but lower than the highest adenosine concentrations in myeloma bone marrow aspirates, which exceeded 150 µM [49]. Further, the Ki value of 38 μM was of the same magnitude as the adenosine levels of about 40 μM observed in experimental brain ischemia [45,46]. Consequently, available data support the assumption that cellular uptake of adenosine analogs is compromised in several illnesses. Importantly, adenosine levels in interstitial fluids and cell surface environments may be significantly higher than in plasma. If so, comparison of plasma adenosine concentrations with K_i_ values for the in vitro adenosine-mediated inhibition of the uptake of adenosine analogs would underestimate a role of adenosine in shaping outcome of treatments with these agents.

Since hypoxia and inflammation with local and systemic release of inflammatory cytokines are main features of the pathophysiology of COVID-19 [61], it is possible that the expression of adenosine transporters is decreased, and the levels of extracellular adenosine are increased, in this disease [62]. Decreased expression of adenosine transporters and high levels of adenosine, which competitively may inhibit cellular uptake of GS-441524, have the potential to reduce the effectiveness of this drug in the treatment of COVID-19. Cellular uptake of GS-441524 in patients with COVID-19 may also be reduced by comorbidities associated with increased extracellular adenosine levels, such as severe cardiovascular disease [63], obesity [64], and cancer [48] in addition to comorbidities or complications of COVID-19 requiring treatment with agents that increase extracellular adenosine levels. Similarly, uptake of GS-441524 after pulmonary delivery, a recently proposed treatment of COVID-19 [11], may be reduced by high levels of adenosine in the bronchoalveolar epithelial lining fluid.

## 4. Intracellular Adenosine Levels

Adenosine kinase (ADK), the primary enzyme responsible for regulation of intracellular levels of adenosine under normal conditions, catalyzes phosphorylation of adenosine and adenosine analogs to their monophosphate derivatives [65]. This enzyme is widely expressed in the human body with a medium level of expression in most organs and tissues under physiological conditions, including the lungs, intestines, liver, kidneys, spleen and lymph nodes [33,66]. ADK is a high-affinity and low-capacity enzyme with a K_m_ of 0.2–0.4 mM, probably operating close to its level of saturation under physiological conditions [65]. The efficiency of the first and likely rate-limiting step in the phosphorylation of adenosine analogs, which eventually leads to the formation of their active antiviral triphosphate derivatives [67], could be highly dependent on intracellular levels of adenosine, since these analogs may have to compete with adenosine for the active site in ADK. In support of this, the in vitro thymidine kinase-mediated phosphorylation of the anti-HIV agent zidovudine has been found to be blocked by thymidine [68].

Under physiological conditions, levels of free cytosolic adenosine appear to be kept in the nanomolar range, thus being comparable to plasma adenosine levels [43]. However, during cellular stress, free cytosolic adenosine levels can significantly increase. Notably, hypoxia increased these levels by 17-fold in guinea pig hearts [69]. Moreover, adenosine levels have been reported to be 2.9-fold higher in peripheral blood mononuclear cells from human subjects with end-stage renal failure requiring hemodialysis than in healthy control subjects [70]. Increased intracellular levels of adenosine in hypoxic tissues may partially result from decreased expression of ADK [71]. The expression of this enzyme is likely also decreased in inflammatory lesions, which often are highly hypoxic [72]. In line with this, expression of ADK has been found to be decreased in human liver cancer [73]. Accordingly, increased intracellular adenosine levels and decrease in the expression of ADK during pathological conditions may lead to markedly decreased phosphorylation of adenosine analogs.

To our knowledge, the affinity of GS-441524 for human ADK has not been reported. GS-441524 differs from adenosine by having C substituted with N at position 4, and substitutions of N with C at positions 7 and 9 in its adenine moiety, in addition to a cyano group linked to the 1′ position in the ribose part (Figure 1). Previously, the adenosine analog tubercidine, in which N at position 7 of its adenine moiety is replaced with C, was reported to be more efficiently phosphorylated than adenosine by mammalian ADKs including human ADK, while substitution of N with C at position 9 led to reduced phosphorylation [74,75]. Moreover, psicofuranine, an antineoplastic and antibiotic adenosine analog with a 1′-hydroxymethyl group, but otherwise identical to adenosine, is not a substrate of rabbit ADK [76]. We are not aware of studies that have examined the impact of substitution at position 4 in adenosine on the phosphorylation by ADK.

Collectively, the available evidence favors the notion that intracellular levels of adenosine are increased in the airways of patients with COVID-19. This may lead to a decrease in the formation of GS-441524 monophosphate by ADK, thus potentially decreasing the levels of GS-441524 triphosphate.

## 5. Anti-SARS-CoV-2 Activity of GS-441524 Beyond the Airways

SARS-CoV-2 is capable of infecting a range of human tissues and organs beyond the upper and lower respiratory tract, including the heart, spleen, liver, gastrointestinal tract, kidneys, lymph nodes, brain and the endothelial lining of the blood vessels [77,78,79,80]. Moreover, this virus has been reported to produce abortive infection of macrophages and dendritic cells, accompanied by expression of proinflammatory cytokines and chemokines [81]. It is likely that variations in the expression of nucleoside transporters and ADK across tissues and organs lead to differences in uptake and phosphorylation of GS-441524, perhaps manifested in different GS-441524 triphosphate levels. Differences in extracellular and intracellular adenosine levels throughout the body may also influence uptake and phosphorylation of GS-441524 and, ultimately, lead to low intracellular levels of GS-441524 triphosphate in some organs and tissues. Accordingly, GS-441524 may be effective in eliminating SARS-CoV-2 from some sites in the body, but not others.

## 6. Concluding Remarks

Low levels of expression of the adenosine transporters in alveolar cells such as ENT1, one of the major adenosine transporters, may limit the potency of GS-441524 in the treatment of COVID-19 pneumonia. It is possible that GS-441524 is more effective against early stages of COVID-19, i.e., before spreading of the virus to the lower airways has occurred, than later stages of the infection, since the majority of the transporters implicated in cellular uptake of adenosine analogs appear to be more abundantly expressed in the upper than lower airways. Similarly, abundant expression of these nucleoside transporters in organs and tissues beyond the airways may lead to high anti-SARS-CoV-2 activity of GS-441524. The potential impact of hypoxia on the outcome of treatment of COVID-19 with GS-441524 appears not to have been considered previously. It is likely that tissue hypoxia and inflammation in COVID-19 leads to decrease in the expression of the adenosine transporters and ADK, in addition to increases in intracellular and extracellular levels of adenosine. Collectively, this could lead to decreased formation of GS-441524 triphosphate and limit the potency of GS-441524 for treatment of COVID-19 and other viral diseases associated with tissue hypoxia and severe inflammatory reactions. Likewise, comorbidities in COVID-19, which are associated with increased adenosine levels, may reduce the potency of GS-441524. Knowledge about the potential impact of hypoxia and inflammation on effectiveness of GS-441524 against COVID-19 may have relevance for treatments with other adenosine analogs.

## Figures and Tables

**Table 2:** Kinetic parameter values for cellular influx of adenosine and analogs of adenosine mediated by human recombinant equilibrative transporters 1 and 2.

Substrate	Kinetic Parameter Values ^1^	
	ENT1 (μM)	ENT2 (μM)
Adenosine	17.8–40	106–140
Clofarabine	108–114	328
Fludarabine	107	168 ^2^
Cladribine	23	50 ^2^
Tecadenoson	24–196	NA

Based on reports by Köse and Schiedel [32] and Lepist et al. [19]. ENT1: equilibrative transporter 1. ENT2: equilibrative transporter 2. ^1^ Kinetic parameters are given as Michaelis constant values unless otherwise stated. ^2^ Inhibitor constant values represent ability to inhibit transporter-mediated adenosine influx.

**Table 3:** Expression of nucleoside transporters potentially implicated in the uptake of GS-441524 in the human respiratory system.

Transporter ^1^	Nasopharynx (Respiratory Epithelial Cells)	Bronchus (Respiratory Epithelial Cells)	Lung ^2^	
			Alveolar Cells (Pneumocytes)	Macrophages
CNT2 (solute carrier family 28 member 2, SLC 28A2)	Not detected	Not detected	Not detected	Not detected
CNT3 (solute carrier family 28 member 3, SLC 28A3)	High	Medium	Not detected	Not detected
ENT1 (solute carrier family 29 member 1, SLC 29A1)	Medium	Medium	Low	Medium
ENT2 (solute carrier family 29 member 2, SLC 29A2)	High	Medium	Medium	Low

^1^ Expression data derived from the Human Protein Atlas [33]. ^2^ Percentage of alveolar cells ranged from 20% to 40% with a mean of about 32% without distinction between alveolar type 1 and type 2 cells in the samples analyzed [33]. Previously, alveolar type 1 and type 2 cells have been found to comprise 8% and 16%, respectively, of the cells in the normal human lung parenchyma, whereas alveolar macrophages accounted for 3% to 19% of the cells in this tissue [35].

## References

[1] Eastman R.T., Roth J.S., Brimacombe K.R., Simeonov A., Shen M., Patnaik S., Hall M.D. (2020). Remdesivir: A Review of Its Discovery and Development Leading to Emergency Use Authorization for Treatment of COVID-19. ACS Cent. Sci..

[2] Beigel J.H., Tomashek K.M., Dodd L.E., Mehta A.K., Zingman B.S., Kalil A.C., Hohmann E., Chu H.Y., Luetkemeyer A., Kline S. (2020). Remdesivir for the Treatment of Covid-19 - Final Report. N. Engl. J. Med..

[3] Siemieniuk R., Rochwerg B., Agoritsas T., Lamontagne F., Leo Y.-S., Macdonald H., Agarwal A., Zeng L., Lytvyn L., Appiah J.A. (2020). A Living WHO Guideline on Drugs for Covid-19. BMJ.

[4] Mehellou Y., Rattan H.S., Balzarini J. (2018). The ProTide Prodrug Technology: From the Concept to the Clinic. J. Med. Chem..

[5] Nies A.T., König J., Hofmann U., Kölz C., Fromm M.F., Schwab M. (2021). Interaction of Remdesivir with Clinically Relevant Hepatic Drug Uptake Transporters. Pharmaceutics.

[6] Cocucci E., Kim J.Y., Bai Y., Pabla N. (2017). Role of Passive Diffusion, Transporters, and Membrane Trafficking-Mediated Processes in Cellular Drug Transport. Clin. Pharmacol. Ther..

[7] Li Y., Cao L., Li G., Cong F., Li Y., Sun J., Luo Y., Chen G., Li G., Wang P. (2021). Remdesivir Metabolite GS-441524 Effectively Inhibits SARS-CoV-2 Infection in Mouse Models. J. Med. Chem..

[8] Pruijssers A.J., George A.S., Schäfer A., Leist S.R., Gralinksi L.E., Dinnon K.H., Yount B.L., Agostini M.L., Stevens L.J., Chappell J.D. (2020). Remdesivir Inhibits SARS-CoV-2 in Human Lung Cells and Chimeric SARS-CoV Expressing the SARS-CoV-2 RNA Polymerase in Mice. Cell Rep..

[9] Agostini M.L., Andres E.L., Sims A.C., Graham R.L., Sheahan T.P., Lu X., Smith E.C., Case J.B., Feng J.Y., Jordan R. (2018). Coronavirus Susceptibility to the Antiviral Remdesivir (GS-5734) Is Mediated by the Viral Polymerase and the Proofreading Exoribonuclease. mBio.

[10] Pedersen N.C., Perron M., Bannasch M., Montgomery E., Murakami E., Liepnieks M., Liu H. (2019). Efficacy and Safety of the Nucleoside Analog GS-441524 for Treatment of Cats with Naturally Occurring Feline Infectious Peritonitis. J. Feline Med. Surg..

[11] Yan V.C., Muller F.L. (2020). Advantages of the Parent Nucleoside GS-441524 over Remdesivir for Covid-19 Treatment. ACS Med. Chem. Lett..

[12] Von Keutz T., Williams J.D., Kappe C.O. (2021). Flash Chemistry Approach to Organometallic C-Glycosylation for the Synthesis of Remdesivir. Org. Process Res. Dev..

[13] Choi J.-S., Berdis A.J. (2012). Nucleoside Transporters: Biological Insights and Therapeutic Applications. Future Med. Chem..

[14] Pastor-Anglada M., Pérez-Torras S. (2018). Emerging Roles of Nucleoside Transporters. Front. Pharmacol..

[15] Young J.D. (2016). The SLC28 (CNT) and SLC29 (ENT) Nucleoside Transporter Families: A 30-Year Collaborative Odyssey. Biochem. Soc. Trans..

[16] Pastor-Anglada M., Pérez-Torras S. (2018). Who Is Who in Adenosine Transport. Front. Pharmacol..

[17] Heyne N., Benöhr P., Mühlbauer B., Delabar U., Risler T., Osswald H. (2004). Regulation of Renal Adenosine Excretion in Humans–Role of Sodium and Fluid Homeostasis. Nephrol. Dial. Transplant..

[18] Eyer L., Nencka R., de Clercq E., Seley-Radtke K., Růžek D. (2018). Nucleoside Analogs as a Rich Source of Antiviral Agents Active against Arthropod-Borne Flaviviruses. Antivir. Chem. Chemother..

[19] Lepist E.-I., Damaraju V.L., Zhang J., Gati W.P., Yao S.Y.M., Smith K.M., Karpinski E., Young J.D., Leung K.H., Cass C.E. (2013). Transport of A1 Adenosine Receptor Agonist Tecadenoson by Human and Mouse Nucleoside Transporters: Evidence for Blood-Brain Barrier Transport by Murine Equilibrative Nucleoside Transporter 1 MENT1. Drug Metab. Dispos..

[20] Davis T.D., Gerry C.J., Tan D.S. (2014). General Platform for Systematic Quantitative Evaluation of Small-Molecule Permeability in Bacteria. ACS Chem. Biol..

[21] Altaweraqi R.A., Yao S.Y.M., Smith K.M., Cass C.E., Young J.D. (2020). HPLC Reveals Novel Features of Nucleoside and Nucleobase Homeostasis, Nucleoside Metabolism and Nucleoside Transport. Biochim. Biophys. Acta Biomembr..

[22] Huang W., Zeng X., Shi Y., Liu M. (2017). Functional Characterization of Human Equilibrative Nucleoside Transporter 1. Protein Cell.

[23] Fernández-Calotti P.X., Colomer D., Pastor-Anglada M. (2011). Translocation of Nucleoside Analogs across the Plasma Membrane in Hematologic Malignancies. Nucleosides Nucleotides Nucleic Acids.

[24] Ritzel M.W.L., Ng A.M.L., Yao S.Y.M., Graham K., Loewen S.K., Smith K.M., Ritzel R.G., Mowles D.A., Carpenter P., Chen X.-Z. (2001). Molecular Identification and Characterization of Novel Human and Mouse Concentrative Na^+^-Nucleoside Cotransporter Proteins (HCNT3 and MCNT3) Broadly Selective for Purine and Pyrimidine Nucleosides (System Cib). J. Biol. Chem..

[25] Homminga I., Zwaan C.M., Manz C.Y., Parker C., Bantia S., Smits W.K., Higginbotham F., Pieters R., Meijerink J.P.P. (2011). In Vitro Efficacy of Forodesine and Nelarabine (Ara-G) in Pediatric Leukemia. Blood.

[26] Hawley S.A., Ross F.A., Russell F.M., Atrih A., Lamont D.J., Hardie D.G. (2020). Mechanism of Activation of AMPK by Cordycepin. Cell. Chem. Biol..

[27] Pastor-Anglada M., Molina-Arcas M., Casado F.J., Bellosillo B., Colomer D., Gil J. (2004). Nucleoside Transporters in Chronic Lymphocytic Leukaemia. Leukemia.

[28] Prus K.L., Averett D.R., Zimmerman T.P. (1990). Transport and Metabolism of 9-*β*- d-Arabinofuranosylguanine in a Human T-Lymphoblastoid Cell Line: Nitrobenzylthioinosine-Sensitive and -Insensitive Influx. Cancer Res..

[29] Robinson-White A.J., Bossis I., Hsiao H.-P., Nesterova M., Leitner W.W., Stratakis C.A. (2009). 8-Cl-Adenosine Inhibits Proliferation and Causes Apoptosis in B-Lymphocytes via Protein Kinase A-Dependent and Independent Effects: Implications for Treatment of Carney Complex-Associated Tumors. J. Clin. Endocrinol. Metab..

[30] Boswell-Casteel R.C., Hays F.A. (2017). Equilibrative Nucleoside Transporters—A Review. Nucleosides Nucleotides Nucleic Acids.

[31] Kim S., Chen J., Cheng T., Gindulyte A., He J., He S., Li Q., Shoemaker B.A., Thiessen P.A., Yu B. (2021). PubChem in 2021: New Data Content and Improved Web Interfaces. Nucleic Acids Res..

[32] Köse M., Schiedel A.C. (2009). Nucleoside/Nucleobase Transporters: Drug Targets of the Future?. Future Med. Chem..

[33] Uhlén M., Fagerberg L., Hallström B.M., Lindskog C., Oksvold P., Mardinoglu A., Sivertsson Å., Kampf C., Sjöstedt E., Asplund A. (2015). Tissue-Based Map of the Human Proteome. Science.

[34] Mulay A., Konda B., Garcia G., Yao C., Beil S., Villalba J.M., Koziol C., Sen C., Purkayastha A., Kolls J.K. (2021). SARS-CoV-2 Infection of Primary Human Lung Epithelium for COVID-19 Modeling and Drug Discovery. Cell Rep..

[35] Crapo J.D., Barry B.E., Gehr P., Bachofen M., Weibel E.R. (1982). Cell Number and Cell Characteristics of the Normal Human Lung. Am. Rev. Respir. Dis..

[36] Borea P.A., Gessi S., Merighi S., Vincenzi F., Varani K. (2018). Pharmacology of Adenosine Receptors: The State of the Art. Physiol. Rev..

[37] Kumar V., Sharma A. (2009). Adenosine: An Endogenous Modulator of Innate Immune System with Therapeutic Potential. Eur. J. Pharmacol..

[38] Harley E.R., Paterson A.R., Cass C.E. (1982). Initial Rate Kinetics of the Transport of Adenosine and 4-Amino-7-(*β*-d-Ribofuranosyl)Pyrrolo[2,3-*d*]Pyrimidine (Tubercidin) in Cultured Cells. Cancer Res..

[39] Prus K.L., Heidenreich A.C., Zimmerman T.P. (1992). Transport of the Anti-Varicella-Zoster Virus Agent 6-Methoxypurine Arabinoside and Its 2′-O-Valerate Prodrug into Human Erythrocytes. Antimicrob. Agents Chemother..

[40] Schulte G., Fredholm B.B. (2003). Signalling from Adenosine Receptors to Mitogen-Activated Protein Kinases. Cell. Signal..

[41] Gaubert M., Marlinge M., Kerbaul F., Resseguier N., Laine M., Cautella J., Cordier C., Colomb B., Kipson N., Thuny F. (2018). Adenosine Plasma Level and A2A Receptor Expression in Patients With Cardiogenic Shock. Crit. Care Med..

[42] Borea P.A., Gessi S., Merighi S., Vincenzi F., Varani K. (2017). Pathological Overproduction: The Bad Side of Adenosine. Br. J. Pharmacol..

[43] Fredholm B.B. (2007). Adenosine, an Endogenous Distress Signal, Modulates Tissue Damage and Repair. Cell Death Differ..

[44] Kumar V. (2018). Inflammasomes: Pandora’s Box for Sepsis. J. Inflamm. Res..

[45] Latini S., Bordoni F., Pedata F., Corradetti R. (1999). Extracellular Adenosine Concentrations during in Vitro Ischaemia in Rat Hippocampal Slices. Br. J. Pharmacol..

[46] Hagberg H., Andersson P., Lacarewicz J., Jacobson I., Butcher S., Sandberg M. (1987). Extracellular Adenosine, Inosine, Hypoxanthine, and Xanthine in Relation to Tissue Nucleotides and Purines in Rat Striatum during Transient Ischemia. J. Neurochem..

[47] Martin C., Leone M., Viviand X., Ayem M.L., Guieu R. (2000). High Adenosine Plasma Concentration as a Prognostic Index for Outcome in Patients with Septic Shock. Crit. Care Med..

[48] Kumar V. (2013). Adenosine as an Endogenous Immunoregulator in Cancer Pathogenesis: Where to Go?. Purinergic Signal..

[49] Horenstein A.L., Quarona V., Toscani D., Costa F., Chillemi A., Pistoia V., Giuliani N., Malavasi F. (2016). Adenosine Generated in the Bone Marrow Niche Through a CD38-Mediated Pathway Correlates with Progression of Human Myeloma. Mol. Med..

[50] Li S., Gong M., Zhao F., Shao J., Xie Y., Zhang Y., Chang H. (2018). Type I Interferons: Distinct Biological Activities and Current Applications for Viral Infection. Cell. Physiol. Biochem..

[51] Niemelä J., Henttinen T., Yegutkin G.G., Airas L., Kujari A.-M., Rajala P., Jalkanen S. (2004). IFN-α Induced Adenosine Production on the Endothelium: A Mechanism Mediated by CD73 (Ecto-5′-Nucleotidase) Up-Regulation. J. Immunol..

[52] Okada S.F., Zhang L., Kreda S.M., Abdullah L.H., Davis C.W., Pickles R.J., Lazarowski E.R., Boucher R.C. (2011). Coupled Nucleotide and Mucin Hypersecretion from Goblet-Cell Metaplastic Human Airway Epithelium. Am. J. Respir. Cell Mol. Biol..

[53] Aeffner F., Woods P.S., Davis I.C. (2014). Activation of A1-Adenosine Receptors Promotes Leukocyte Recruitment to the Lung and Attenuates Acute Lung Injury in Mice Infected with Influenza A/WSN/33 (H1N1) Virus. J. Virol..

[54] Driver A.G., Kukoly C.A., Ali S., Mustafa S.J. (1993). Adenosine in Bronchoalveolar Lavage Fluid in Asthma. Am. Rev. Respir. Dis..

[55] Grenz A., Homann D., Eltzschig H.K. (2011). Extracellular Adenosine: A Safety Signal That Dampens Hypoxia-Induced Inflammation During Ischemia. Antioxid. Redox. Signal..

[56] Bowser J.L., Lee J.W., Yuan X., Eltzschig H.K. (2017). The Hypoxia-Adenosine Link during Inflammation. J. Appl. Physiol..

[57] Mayati A., Moreau A., Jouan E., Febvre-James M., Denizot C., Parmentier Y., Fardel O. (2018). mRNA Expression and Activity of Nucleoside Transporters in Human Hepatoma HepaRG Cells. Pharmaceutics.

[58] Visser F., Vickers M.F., Ng A.M.L., Baldwin S.A., Young J.D., Cass C.E. (2002). Mutation of Residue 33 of Human Equilibrative Nucleoside Transporters 1 and 2 Alters Sensitivity to Inhibition of Transport by Dilazep and Dipyridamole. J. Biol. Chem..

[59] Kitakaze M., Minamino T., Node K., Koretsune Y., Komamura K., Funaya H., Kuzuya T., Hori M. (1998). Elevation of Plasma Adenosine Levels May Attenuate the Severity of Chronic Heart Failure. Cardiovasc. Drugs Ther..

[60] Capecchi P.L., Rechichi S., Lazzerini P.E., Collini A., Guideri F., Ruggieri G., Carmellini M., Laghi-Pasini F. (2005). Cyclosporin and Tacrolimus Increase Plasma Levels of Adenosine in Kidney Transplanted Patients. Transpl. Int..

[61] Serebrovska Z.O., Chong E.Y., Serebrovska T.V., Tumanovska L.V., Xi L. (2020). Hypoxia, HIF-1α, and COVID-19: From Pathogenic Factors to Potential Therapeutic Targets. Acta Pharmacol. Sin..

[62] Abouelkhair M.A. (2020). Targeting Adenosinergic Pathway and Adenosine A2A Receptor Signaling for the Treatment of COVID-19: A Hypothesis. Med. Hypotheses.

[63] Gariboldi V., Vairo D., Guieu R., Marlingue M., Ravis E., Lagier D., Mari A., Thery E., Collart F., Gaudry M. (2017). Expressions of Adenosine A2A Receptors in Coronary Arteries and Peripheral Blood Mononuclear Cells are Correlated in Coronary Artery Disease Patients. Int. J. Cardiol..

[64] Escudero A., Carreño B., Retamal N., Celis C., Castro L., Aguayo C., Acurio J., Escudero C. (2012). Elevated Concentrations of Plasma Adenosine in Obese Children. Biofactors.

[65] Boison D. (2013). Adenosine Kinase: Exploitation for Therapeutic Gain. Pharmacol. Rev..

[66] The UniProt Consortium (2019). UniProt: A Worldwide Hub of Protein Knowledge. Nucleic Acids Res..

[67] Van Rompay A., Johansson M., Karlsson A. (2000). Phosphorylation of Nucleosides and Nucleoside Analogs by Mammalian Nucleoside Monophosphate Kinases. Pharmacol. Ther..

[68] Furman P.A., Fyfe J.A., Clair M.H.S., Weinhold K., Rideout J.L., Freeman G.A., Lehrman S.N., Bolognesi D.P., Broder S., Mitsuya H. (1986). Phosphorylation of 3′-Azido-3′-Deoxythymidine and Selective Interaction of the 5′-Triphosphate with Human Immunodeficiency Virus Reverse Transcriptase. Proc. Natl. Acad. Sci. USA.

[69] Decking U.K., Schlieper G., Kroll K., Schrader J. (1997). Hypoxia-Induced Inhibition of Adenosine Kinase Potentiates Cardiac Adenosine Release. Circ. Res..

[70] Sampol J., Dussol B., Fenouillet E., Capo C., Mege J.L., Halimi G., Bechis G., Brunet P., Rochat H., Berland Y. (2001). High Adenosine and Deoxyadenosine Concentrations in Mononuclear Cells of Hemodialyzed Patients. J. Am. Soc. Nephrol..

[71] Morote-Garcia J.C., Rosenberger P., Kuhlicke J., Eltzschig H.K. (2008). HIF-1-Dependent Repression of Adenosine Kinase Attenuates Hypoxia-Induced Vascular Leak. Blood.

[72] Eltzschig H.K., Carmeliet P. (2011). Hypoxia and Inflammation. N. Engl. J. Med..

[73] El-Kharrag R., Owen R., Boison D. (2019). Adenosine Kinase Deficiency Increases Susceptibility to a Carcinogen. J. Caffeine Adenosine Res..

[74] McGaraughty S., Cowart M., Jarvis M.F. (2001). Recent Developments in the Discovery of Novel Adenosine Kinase Inhibitors: Mechanism of Action and Therapeutic Potential. CNS Drug Rev..

[75] Schnebli H.P., Hill D.L., Bennett L.L. (1967). Purification and Properties of Adenosine Kinase from Human Tumor Cells of Type H. Ep. No. 2. J. Biol. Chem..

[76] Lindberg B., Klenow H., Hansen K. (1967). Some Properties of Partially Purified Mammalian Adenosine Kinase. J. Biol. Chem..

[77] Feng Z., Diao B., Wang R., Wang G., Wang C., Tan Y., Liu L., Wang C., Liu Y., Liu Y. (2020). The Novel Severe Acute Respiratory Syndrome Coronavirus 2 (SARS-CoV-2) Directly Decimates Human Spleens and Lymph Nodes. medRxiv.

[78] Remmelink M., De Mendonça R., D’Haene N., De Clercq S., Verocq C., Lebrun L., Lavis P., Racu M.-L., Trépant A.-L., Maris C. (2020). Unspecific Post-Mortem Findings despite Multiorgan Viral Spread in COVID-19 Patients. Crit. Care.

[79] Varga Z., Flammer A.J., Steiger P., Haberecker M., Andermatt R., Zinkernagel A.S., Mehra M.R., Schuepbach R.A., Ruschitzka F., Moch H. (2020). Endothelial Cell Infection and Endotheliitis in COVID-19. Lancet.

[80] Xiao F., Tang M., Zheng X., Liu Y., Li X., Shan H. (2020). Evidence for Gastrointestinal Infection of SARS-CoV-2. Gastroenterology.

[81] Zheng J., Wang Y., Li K., Meyerholz D.K., Allamargot C., Perlman S. (2021). Severe Acute Respiratory Syndrome Coronavirus 2-Induced Immune Activation and Death of Monocyte-Derived Human Macrophages and Dendritic Cells. J. Infect. Dis..

